# Crystallographic insights into lipid-membrane protein interactions in microbial rhodopsins

**DOI:** 10.3389/fmolb.2024.1503709

**Published:** 2024-11-07

**Authors:** S. Bukhdruker, I. Melnikov, C. Baeken, T. Balandin, V. Gordeliy

**Affiliations:** ^1^ Institut de Biologie Structurale J.-P. Ebel, Université Grenoble Alpes-CEA-CNRS, Grenoble, France; ^2^ Structural Biology Group, European Synchrotron Radiation Facility, Grenoble, France; ^3^ Institute of Biological Information Processing (IBI-7: Structural Biochemistry), Forschungszentrum Jülich GmbH, Jülich, Germany; ^4^ JuStruct: Jülich Center for Structural Biology, Forschungszentrum Jülich GmbH, Jülich, Germany

**Keywords:** membrane proteins, X-ray crystallography, annular lipids, detergent, lipid cubic phase, bicelles, membrane fusion, oligomerization

## Abstract

The primary goal of our work is to provide structural insights into the influence of the hydrophobic lipid environment on the membrane proteins (MPs) structure and function. Our work will not cover the well-studied hydrophobic mismatch between the lipid bilayer and MPs. Instead, we will focus on the less-studied direct molecular interactions of lipids with the hydrophobic surfaces of MPs. To visualize the first layer of amphiphiles surrounding MPs and analyze their interaction with the proteins, we use the available highest-quality crystallographic structures of microbial rhodopsins. The results of the structure-based analysis allowed us to formulate the hypothetical concept of the role of the nearest layer of the lipids as an integral part of the MPs that are important for their structure and function. We then discuss how the lipid-MPs interaction is influenced by exogenous hydrophobic molecules, noble gases, which can compete with lipids for the surface of MPs and can be used in the systematic approach to verify the proposed concept experimentally. Finally, we raise the problems of currently available structural data that should be overcome to obtain a more profound picture of the lipid-MP interactions.

## Introduction

Lipid-membrane protein (MP) interactions are known to predetermine the MP structure. The alteration of the lipid composition surrounding MPs influences their function (Levental and Lyman, 2022). One of the most studied types of interaction is the one that occurs due to a mismatch between the hydrophobic interacting surfaces of the lipid bilayer and MP. This phenomenon is described in several original works and reviews (Killian, 1998), and it will not be included in the scope of our work.

In contrast, there is a lack of experimental information and analysis on the direct interaction between lipid hydrocarbon chains and the hydrophobic surface of an MP at a molecular level in the absence of the hydrophobic mismatch. There were extensive studies of the general properties of such interactions. In the 1970s, ESR studies resulted in the conclusion that such interactions led to the creation of the layer of the so-called annular lipids around MPs. This may point towards a strong interaction of the nearest layer of hydrophobic sidechains with the hydrophobic MP surface (Jost et al., 1973; Stier and Sackmann, 1973). However, NMR studies that were done later quite compromised the concept of annular lipids (Brown et al., 1977; Oldfield et al., 1978; Skelig and Seklig, 1978). At present, there are different points of view on the existence and functional importance of annular lipids (Gómez-Fernández and Goñi, 2022).

One of the origins of this controversy is that interpreting the results of ESR, NMR, and other experimental studies is not trivial. In addition, there is no systematic analysis of these interactions at the molecular/atomic level. It would be important to visualize directly such annular lipids to prove (or disprove) their existence. The problem here is high-resolution structural data obtained in a controlled experimental environment, which is usually unavailable. Nevertheless, several high-quality structural data sets have been obtained in the past decade, particularly regarding microbial rhodopsins (MRs) across all domains of life (Gordeliy, 2022). These data provide a unique opportunity for a systematic molecular analysis of MP surface interactions with the nearest lipids, which we present in this work.

We have to note that our work does not pretend to be a complete analysis of such lipid-MP interactions. We are rather restricted to the structural analysis of how lipid chains fit the landscape of hydrophobic membrane surface, how it depends on the origin of MRs and lipids (we will demonstrate this in the example of archaeal and bacterial lipids), and how the surrounding lipids influence the structure and dynamics of MRs. Nevertheless, we hope that it helps us better understand what is known and what should be done in the future to analyze such interactions. In particular, we demonstrate that precise knowledge of the distances between surface atoms of MRs and atoms of lipid chains nearest to this surface is important to understand how this interaction influences the MR structure and function. Van der Waals forces decay quickly with distance, and even small gaps between certain parts of the MR surface and the corresponding atoms of lipid chains may lead to disorder of the lipid chains and influence the stability or local dynamics of MR. We conclude that it will be important to construct future experiments so that the proteins are surrounded with native lipids, while the non-native ones could be used to probe these lipid-MP interactions.

This work is partially encouraged by our recent structural study of the interaction between noble gases and MRs, in which we showed numerous atoms of noble gas bound to their hydrophobic surface (Melnikov et al., 2022). The atoms compete with lipids for access to the hydrophobic MP surface and can be used to probe lipid-MP interactions.

We believe that our findings may help to provide a guide to comprehensive studies of such interactions at a molecular/atomic level. This will be discussed in the final part of our paper.

## Main part

### Lipids and amphiphiles in high-resolution crystal structures of *Hs*BR

**FIGURE 1:**
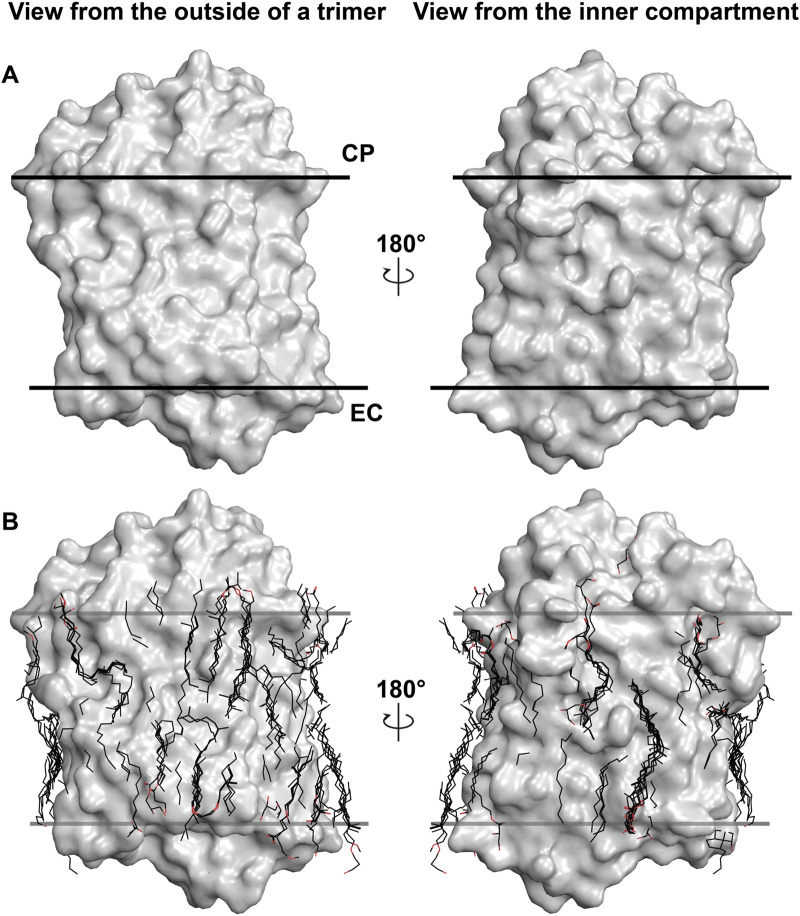
Lipids in high-resolution crystal structures of *Hs*BR. **(A)**, van der Waals surface in the crystal structure of *Hs*BR [PDB ID:7Z09 (Borshchevskiy et al., 2022)]. **(B)**, lipids in the crystal structures of *Hs*BR with a resolution higher than 2 Å. Lipids within 5 Å of the protomer are shown as sticks. **(A, B)**, the structure is shown from two views: a view from the outside of the trimer and from the inner compartment. The calculated hydrophobic-hydrophilic boundaries (Lomize et al., 2012) are shown as lines.

To demonstrate the importance of lipids on the MR structure, we should refer to Figure 1. The figure shows the most studied MR ― an archaeal light-driven proton pump from *Halobacterium salinarum* named bacteriorhodopsin (*Hs*BR) in the lipid environment. Most of the data in this paper is based on the studies of *Hs*BR and its closest homologs. The panel A of the figure shows the van der Waals surface of the highest-resolution crystal structure of *Hs*BR available [1.05 Å-resolution, P6_3_ space group, 1 molecule in asymmetric unit, ASU; protein data bank accession number, PDB ID:7Z09 (Borshchevskiy et al., 2022)]. We can recognize that the protein’s surface is fully covered with crevices dedicated to lipids (Figure 1A). What happens if we then align all the unique published structures of *Hs*BR with a resolution higher than 2 Å? In that case, we will see that despite the crystallization methods, the resolved hydrophobic sidechains of the lipids are trying to fit into the crevices (Figure 1B). In certain instances, when the lipid is not native to the protein, its structure will not allow it to fit perfectly, resulting in the discordance of certain parts of the lipids. As we will show later in the text, this can have crucial consequences for the function of MRs. Thus, for structural-functional studies of MRs, we must understand what lipids we are working with and correctly model them in the structures. But before discussing the concrete examples, we need to examine which lipids can be observed in crystallographic structures and why it is challenging to determine the origin of the lipids in certain cases.

### What lipids and amphiphiles are observed in crystal structures of MRs

#### Lipids of *Hs*BR

**FIGURE 2:**
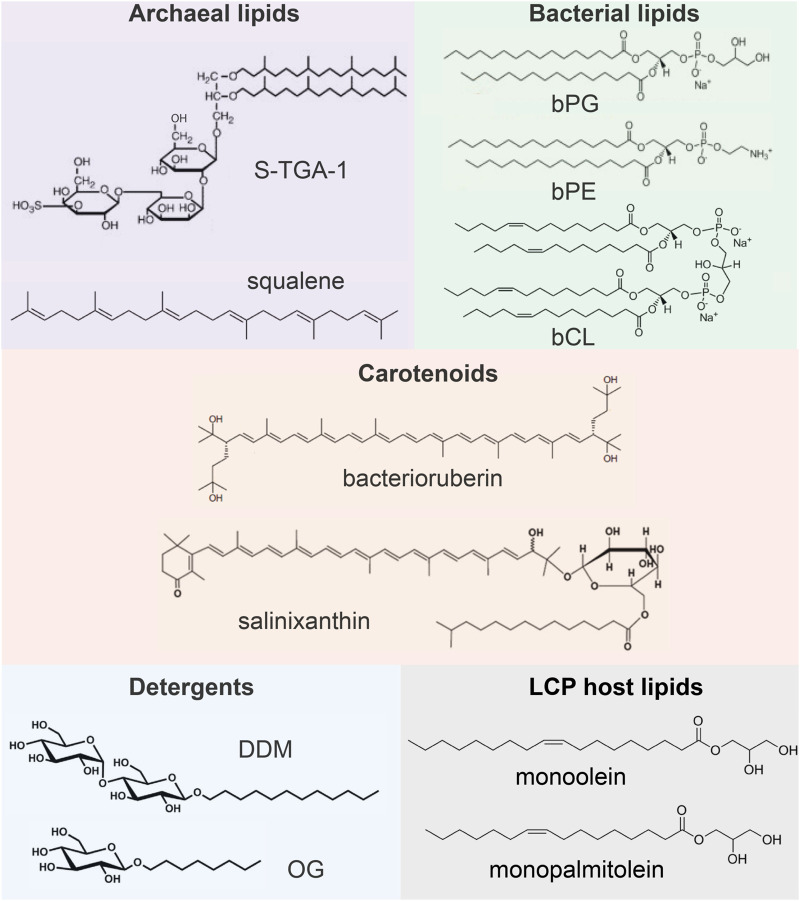
Examples of lipids and other molecules that can be found in the crystal structures of MRs.

In this paragraph, we briefly review what lipids are observed in the available crystal structures of MRs. The information is summarized in Figure 2. In archaea, *Hs*BR is organized in 2D clusters, commonly known as purple membranes (PMs), facilitating phototrophy (Oesterhelt and Stoeckenius, 1971). PM lipids consist of 90% polar and 10% neutral lipids, which are pre-dominantly squalene (Kates, 1993; Renner et al., 2005; Kates et al., 1982). The polar lipids are archaeal phosphatidyl-glycerol (PG), phosphatidyl-glycerol phosphate (PGP), phosphatidyl-glycerol phosphate methylester (PGP-Me), phosphatidyl-glycerol sulfate (PGS), triglycosyldiether (TGD), sulfated triglycoside lipid (S-TGA-1) and sulfated tetraglycosyl diphytanyl-glycerol (S-TeGa). These lipids comprise one-fourth of the PM and significantly affect *Hs*BRs function (Sternberg et al., 1992).

#### Composition of archaeal and bacterial membranes

The composition of membranes of other archaea can be different from PM. Still, it is known that a major difference from bacterial lipids (as well as from synthetic lipids used for crystallization) is that archaeal lipids consist of branched phytanyl sidechains (Caforio and Driessen, 2017; Mencía, 2020). Due to their special chemical structure, such sidechains are clearly visible in crystal structures. However, distinguishing archaeal lipids from each other requires knowledge of their polar heads, which is usually a challenging task for crystallography and often can be done using complementary methods, such as mass spectrometry (MS).

Some of the MRs discussed here have bacterial origin. Typical examples of bacterial lipids are phosphatidyl-glycerol (bPG), phosphatidyl-ethanolamine (bPE), and cardiolipin (bCL) (Horne et al., 2020). The linear acyl groups of these lipids can be easily confused with synthetic lipids, detergents, and polyethylene glycol (PEG) molecules from the crystallization matrix. For this reason, resolution of the lipid polar head is necessary to confirm the nature of the lipid.

Finally, one can find other auxiliary native amphiphilic or hydrophobic molecules in the structures of MRs, e.g., carotenoids [bacterioruberin, salinixanthin (Grivard et al., 2022)], the role of which may vary from MRs stabilization (Yoshimura and Kouyama, 2008) to light harvesting (Balashov et al., 2005; Luecke et al., 2008; Chazan et al., 2023). Such lipids have a very distinctive structure and manifest themselves not only in crystal structure but also in functional tests with these MRs.

#### Different crystallization approaches influence the composition of the hydrophobic layer

Here, we briefly describe currently available MP crystallization methods to understand better, why crystallization protocol is important for preserving native lipids. To extract MRs from their membranes (the process called solubilization), detergent molecules are used (for MRs, these are often n-octyl-β-D-glucoside, or OG, and n-dodecyl-β-D-maltoside, or DDM) which make MRs water soluble by surrounding their hydrophobic surface (Kalipatnapu and Chattopadhyay, 2005). Then, different crystallization approaches with the solubilized protein can be used. MRs can be directly crystallized from detergent (*in surfo* methods) using techniques developed for water-soluble proteins. While this method is the simplest one, it does not always provide crystals of sufficient quality, as detergent may negatively affect the stability of MRs (Lee et al., 2022) and, in some cases, prevent the formation of strong crystalline contacts (Ishchenko et al., 2017).

Several methods were developed to overcome these problems, the general idea of which is to return the MP to the lipid environment. The lipid cubic phase (LCP) or, *in meso*, crystallization (Landau and Rosenbusch, 1996) remarkably advanced the structural biology of MRs, and today, most of the crystal structures of MPs are obtained using this method (Ishchenko et al., 2017). Briefly, in *in meso* method, solubilized MR is mixed with a synthetic host lipid (typically, monoolein, or MO, and monopalmitolein), and the resultant phase (called LCP) is used for the crystallization. Alternatively, the solubilized protein can be reconstituted to bicelles (Faham and Bowie, 2002; Murugova et al., 2022) or other lipid-detergent systems supporting crystallization (Ishchenko et al., 2017; Gourdon et al., 2011).

Finally, in some cases, it is possible to preserve the lipid environment by avoiding the solubilization step, like for membrane fusion crystallization (Takeda et al., 1998), during which the membranes are treated with detergent and the resultant vesicles are used for crystallization. All these methods can be used for stand-alone crystallization or in combination with others.

In the next part of our work, we aim to consider different crystallization techniques for revealing lipid molecules at the MP surface. We would like to address the question: do we really see native lipids in the available structures of MRs, and what are the reasons why only some, but not all, lipids could be resolved? To address this question, we will consider and analyze the lipids at the surfaces of *Hs*BR and other MRs that were obtained by various crystallization techniques (Table 1). Importantly, at this step of the analysis, we only consider for the analysis those available structures for which crystallographic electron densities clearly confirm the nature of the lipid or for which additional data are provided that, in addition to the structural data, indicate the nature of the lipid.

**TABLE 1:** Structures of MRs used for the analysis of lipid-MP interactions.

Protein	Native organism	PDB ID and reference	Expression system	Crystallization method	Detergents or auxiliary hydrophobic/amphiphilic molecules used at any stage of protein handling	Resolution, Å	Oligomeric state	Native lipids, amphiphiles, and hydrophobic molecules in the structure	Other lipids or long-chain molecules
*Hs*BR	*H. salinarum*	2BRD (Grigorieff et al., 1996)	Native	PM structure	Detergents: OG, dodecyltrimethyl ammonium chloride	3.5	Trimer	10 AUN	ND
		1BRR (Essen et al., 1998)	Native	*In surfo*	Detergents: OG	2.9	Trimer	2/3 S-TGA-1, 5/3 AUN	1/3 OG
		1C3W (Luecke et al., 1999)	Native	*In meso*	Detergents: OG Lipids: MO	1.55	Trimer	1 squalene, 9 AUN	4 BAD
		1QHJ (Belrhali et al., 1999)	Native	*In meso*	Detergents: OG Lipids: MO	1.9	Trimer	9 AUN	ND
		3NS0 (Borshchevskiy et al., 2011)	Native	*In meso*	Detergents: 5-cyclohexyl-1-pentyl-β-D-maltoside (CYMAL-5), OG Lipids: MO	1.78	Trimer	ND	9 BAD
		4MD2 (Borshchevskiy et al., 2014)	Native	*In meso*	Detergents: CYMAL-5, OG Lipids: MO	1.73	Trimer	ND	19 BAD
		7Z09 (Borshchevskiy et al., 2022)	Native	*In meso*	Detergents: CYMAL-5, OG Lipids: MO	1.05	Trimer	ND	28 BAD
		1KME (Faham and Bowie, 2002)	Native	Bicelle	Detergents: OG, Chapso Lipids: DMPC	2.0	Monomer	ND	1 BAD
		1BM1 (Sato et al., 1999)	Native	Membrane fusion	Detergents: Tween-20, n-octyl-β-D-thioglucoside (OTG)	3.5	Trimer	1 AUN	ND
		1QM8 (Takeda et al., 1998)	Native	Membrane fusion	Detergents: Tween-20, OTG	2.5	Trimer	1 S-TGA-1, 4 AUN	ND
		1IW6 (Matsui et al., 2002)	Native	Membrane fusion	Detergents: Tween-20, OTG	2.3	Trimer	1 S-TGA-1, 4 AUN	ND
		2ZZL (Yamamoto et al., 2009)	Native	Membrane fusion	Detergents: Tween-20, OTG	2.03	Trimer	1 S-TGA-1, 4 AUN	1 OTG
		4XXJ (Bratanov et al., 2015)	*E. coli*	*In meso*	Detergents: DDM Lipids: MO	1.9	Trimer	ND	12 BAD
*Hs*HR	*H. salinarum*	1E12 (Kolbe et al., 2000)	Native	*In meso*	Detergents: OG Lipids: MO Other: cholate	1.8	Trimer	ND	11 BAD
Arch3	*H. sodomense*	6S6C (Bada Juarez et al., 2021)	Native	*In meso*	Lipids: MO Other: PEG 600	1.07	Monomer	ND	11 BAD
XR	*S. ruber*	3DDL (Luecke et al., 2008)	Native	Bicelle	Detergents: DDM, n-nonyl-β-D-maltoside Lipids: DMPC Other: ethylene glycol	1.9	Monomer	1 salinixanthin	1 DMPC
Arch1	*Halorubrum* sp. *aus-1*	1UAZ (Enami et al., 2006)	Native	Membrane fusion	Detergents: OTG Other: heptane-triol	3.4	Monomer	ND	ND
Arch2	*Halorubrum* sp. *aus-2*	1VGO (Enami et al., 2006)	Native	Membrane fusion	Detergents: Tween-20, n-nonyl-β-D-glucoside (NG)	2.5	Monomer	ND	6 NG
		2EI4 (Yoshimura and Kouyama, 2008)	Native	Membrane fusion	Detergents: Tween-20 (low concentration), NG	2.1	Trimer	1 bacterioruberin, 1 AUN	ND
		2Z55 (Yoshimura and Kouyama, 2008)	Native	Membrane fusion	Detergents: Tween-20 (low concentration), NG	2.5	Trimer	1 bacterioruberin, 1 AUN	ND
		3WQJ (Kouyama et al., 2014)	Native	Membrane fusion	Detergents: Tween-20 (low concentration), NG	1.8	Trimer	1 squalene, 1 bacterioruberin, 4 AUN	ND
*Np*HR	*N*. *pharaonis*	3A7K (Kouyama et al., 2010)	Native	Membrane fusion	Detergents: Tween-20, NG	2.0	Trimer	1 bacterioruberin, 6 AUN	ND
DR3	*H*. *thermotolerans*	4FBZ (Zhang et al., 2013)	*H. salinarum* MPK409	Membrane fusion	Detergents: NG	2.7	Trimer	1 squalene, 1 bacterioruberin, 1 AUN	2 NG
CR3	*H. vallismortis*	4JR8 (Chan et al., 2014)	*H. salinarum* MPK409	Membrane fusion	Detergents: NG	2.3	Trimer	1 bacterioruberin	ND
		4L35 (Chan et al., 2014)	*H. salinarum* MPK409	Membrane fusion	Detergents: NG	2.1	Trimer	1 bacterioruberin	ND
*Hm*BR	*H*. *marismortui*	4PXK (Shevchenko et al., 2014)	*E. coli*	*In meso*	Detergents: DDM Lipids: MO	2.5	Trimer	ND	19 BAD
*Hw*BR	*H. walsbyi*	4QI1 (Hsu et al., 2015)	*E. coli*	*In meso*	Detergents: DDM, n-decyl-β-D-maltoside (DM) Lipids: MO Other: PEG 400	1.85	Trimer	ND	3 BAD
		4QID (Hsu et al., 2015)	*E. coli*	*In meso*	Detergents: DDM, DM Lipids: MO Other: PEG 200	2.57	Anti-parallel dimer	ND	1/2 BAD
		5KKH (Broecker et al., 2016)	*E. coli*	*In meso*	Detergents: DDM, OG Lipids: MO Other: PEG 3350	2.13	Trimer	ND	3 BAD
		5ITE (Broecker et al., 2017)	*E. coli*	*In meso*	Detergents: DDM, OG Lipids: MO Other: PEG 3350	2.18	Trimer	ND	3 BAD
		5ITC (Broecker et al., 2017)	*E. coli*	*In meso*	Lipids: MO, DMPC Other: SMA	2.0	Trimer	ND	10/3 BAD

ND, not detected; AUN, archaeal of unknown type; BAD, linear hydrophobic sidechain of bacterial lipid, auxiliary lipid, or detergent. The number of lipids and amphiphiles in the structures is recalculated per one protomer.

### Lipids from the PM structure of *Hs*BR

*Hs*BRs are organized in PM as 2D crystals, allowing the determination of its structure with the electron diffraction method without the solubilization step [PDB ID: 2BRD (Grigorieff et al., 1996), 1AT9 (Kimura et al., 1997), 1FBB, and 1FBK (Subramanlam and Henderson, 2000)]. Despite the moderate resolution of the data (3.5 Å), the Henderson group reported for the first time the presence of electron densities for six lipids out of ten possible (Grigorieff et al., 1996). This indicated that at least some of the lipids are likely responsible for the integrity of the two-dimensional crystal lattice. Moreover, they found that most of the lipids were resolved near their extracellular portion and suggested that this was probably due to the greater mobility of the cytoplasmic portion associated with protein function.

### *Hs*BR crystallized in detergent

**FIGURE 3:**
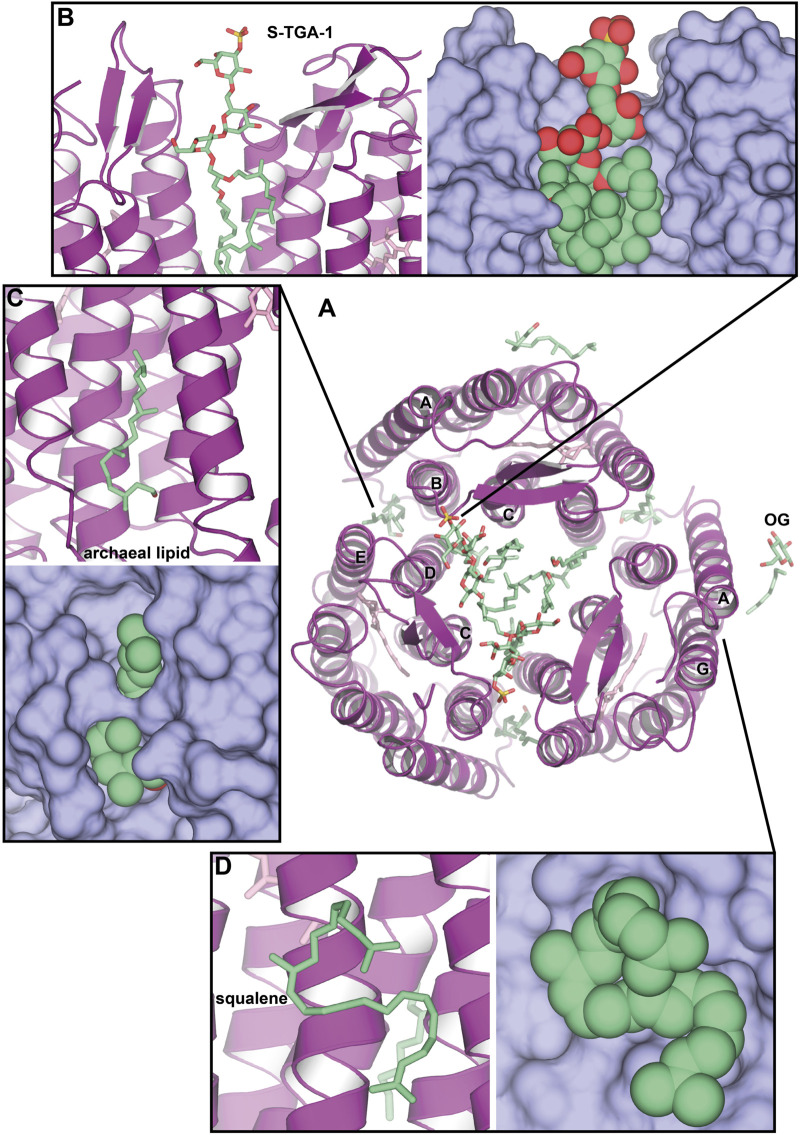
Native lipids in the crystal structures of *Hs*BR. **(A)**, Overall structure of benzamidine-crystallized *Hs*BR [PDB ID: 1BRR (Essen et al., 1998)], a view from the extracellular side. Besides multiple archaeal lipids (shown as green-colored sticks), at least one OG molecule is present in the structure. **(B)**, sulfated triglycoside (S-TGA-1) lipid. **(C)**, uncharacterized archaeal lipid between helices A and B of one protomer and D and E of another. **(D)**, the position of a squalene lipid, found in the LCP-crystallized *Hs*BR structure [PDB ID: 1C3W (Luecke et al., 1999)]: between helices A and F, but not in the benzamidine-crystallized *Hs*BR.

An interesting case of *Hs*BR crystallization was presented by Schertler et al. (1993). In the experiment, benzamidine crystals were used as a nucleation surface for the crystallization of the solubilized *Hs*BR (Figure 3A). Resultant crystals in the C2 space group [PDB ID: 1BRR (Essen et al., 1998)] were diffracting to 2.9 Å-resolution and contained trimers of *Hs*BR in ASU that were packed in a honeycomb-like lattice, different, compared to native packing in PM. What was surprising is that despite the harsh crystallization procedure with the utilization of the solubilized protein and the relatively moderate resolution of the model, 2*m*Fo-*D*Fc maps clearly show the presence of two S-TGA-1 lipids in the inner compartment of the trimers (Figure 3B). The resolution of the lipids was likely possible due to C2 crystal packing, where the polar lipid head was stabilized by the H-bonds with either the mainchain of the β-sheet (G72, G73, and E73) or the S-TGA-1 lipid of the *Hs*BR molecule, belonging to the other bilayer. Another uncharacterized archaeal lipid is bound between the helices A and B of one protomer and D and E of another (Figure 3C). Both these lipids are likely involved in stabilizing the trimer. The authors used negative-ion nanoESI-MS to confirm that lipids from PM were present in crystals. Yet, we can see at least one OG molecule in the structure (Figure 3A), meaning that part of the lipids was gone. This example demonstrates the preservation of native lipids, presumably important for the structure and function of the protein. It is also shown that crystal packing may influence the observations.

### Lipid cubic phase structures

#### Squalene in *Hs*BR structure

One of the earliest LCP high-resolution structures of *Hs*BR from relatively low-twinned crystals (1.55 Å-resolution) identified a squalene molecule (Figure 3D), a specific native PM lipid, bound to the groove near the helix G [PDB ID: 1C3W (Luecke et al., 1999)]. The groove formed by hydrophobic residues L19, L22, V210, V213, V217, and L221, as well as by the sidechain of S214, can accommodate a highly curved squalene molecule. This, as well as the fact that squalene is bound near the retinal-Schiff base (RSB) region and is known to participate in RSB reprotonation by D96 during the photocycle, points towards the preference of the protein surface to certain lipids. Throughout this paper, we will show that this might also be the case for other lipids. The work reported 13 other lipid chain fragments, but they were not characterized in detail.

#### Identification of other native lipids in the LCP structure of *Hs*BR

The first high-resolution LCP-crystallized *Hs*BR obtained with non-twinned crystals [1.9 Å-resolution, PDB ID: 1QHJ (Belrhali et al., 1999)] revealed nine lipids surrounding the protein, which were also studied by MS. Six of the lipids were located within the symmetrical trimers, one ― in the central compartment of each trimer, while the remaining two lipids fastened the monomers together. The authors observed that the alkyl sidechains of lipids had multiple van der Waals contacts with the protein surface.

The head groups of the lipids were not identified, so the UV-MALDI-MS was applied to resolve the observed lipids. The lipase-treated crystals (Nollert and Landau, 1998) showed peaks for PGP-Me, TGD, and S-TGA-1 lipids while lacking peaks from S-TeGa and PG. This confirms that some PM lipids are conserved at the *Hs*BR surface despite solubilization and LCP crystallization. This means that they interact quite strongly with the surface of *Hs*BR. It is presumably because they adapt perfectly to the landscape of the proteins’ surface.

#### What true-atomic resolution adds to our understanding of lipid-MP interaction

**FIGURE 4:**
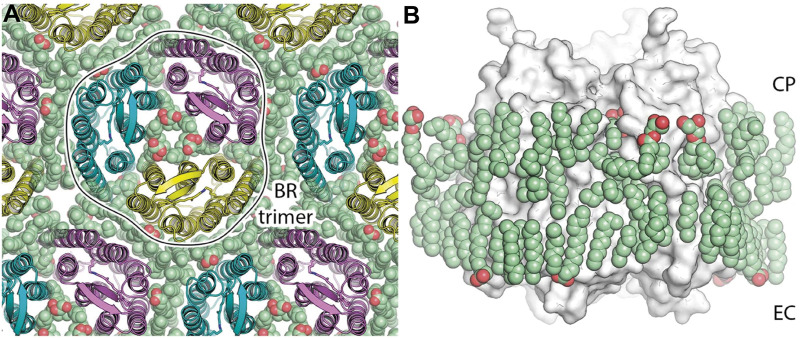
*Hs*BR trimer and lipid molecules in the true-atomic-resolution structure. **(A)** a view on the crystal monolayer from the extracellular side (EC) of *Hs*BR [PDB ID: 7Z09 (Borshchevskiy et al., 2022)]. The trimer of *Hs*BR (cartoon representation) is contoured for clarity. Lipid molecules are shown as spheres and colored green. **(B)**, a side view of the *Hs*BR trimer (white surface) surrounded by the ring of lipid molecules (green spheres). Figure adapted from Borshchevskiy et al. (2022).

Recently, a 1.05 Å-resolution crystal structure of *Hs*BR was published, which allowed us to fix the positions of a record number of lipid fragments, 28 per protomer, in the structure [PDB ID: 7Z09 (Borshchevskiy et al., 2022)]. Numerous lipid chains almost entirely cover the hydrophobic region of *Hs*BR (Figure 4). Unfortunately, no polar lipid heads could be resolved in the structure. Also, the electron densities do not contain signs of methyl branching. That might indicate that the native lipids were disturbed or even displaced by MO from the crystallization matrix and/or detergent. Nevertheless, even if the latter is true, the positioning of the amphiphiles in the crevices very accurately conveys the lipidic environment of native PM. For example, we can look at the extracellular part of the central compartment. Putative MO molecules in the true-atomic resolution structure resemble native S-TGA-1 lipids, except that the former have linear sidechains and no extended polar heads.

Another example of a true-atomic-resolution structure would be Archaerhodopsin-3 from *Halorubrum sodomense* (Arch3) (Bada Juarez et al., 2021). The crystals (PDB ID: 6S6C) diffracted to 1.07 Å-resolution, were in the P2_1_2_1_2_1_ space group, and contained 1 molecule in ASU. 11 lipid fragments were modeled in the structure, none of which belonging to archaea, as indicated by the absence of isoprenoid chains. It is worth noting that, unlike *Hs*BR, Arch3 was crystallized in the monomeric form, which indicates that the disturbance/absence of native lipids could have induced, in this case, the breakdown of oligomers.

### Structure of *Hs*BR and xanthorodopsin crystallized from bicelles

Bicelle crystallization was developed as an alternative to detergent and LCP crystallization to combine the advantages of both methods (Faham and Bowie, 2002; Murugova et al., 2022). However, the crystal structure of initially solubilized *Hs*BR obtained by the method (P2_1_ space group, 2 Å-resolution, 2 molecules in ASU, PDB ID: 1KME) lacked most of the archaeal lipids (only two lipid fragments were modeled for two *Hs*BR molecules). Moreover, *Hs*BR lost its oligomeric organization and, instead, was crystallized as monomers. It seemed like the protein was stripped of most of its native lipids, leading to the disintegration of the trimers.

**FIGURE 5:**
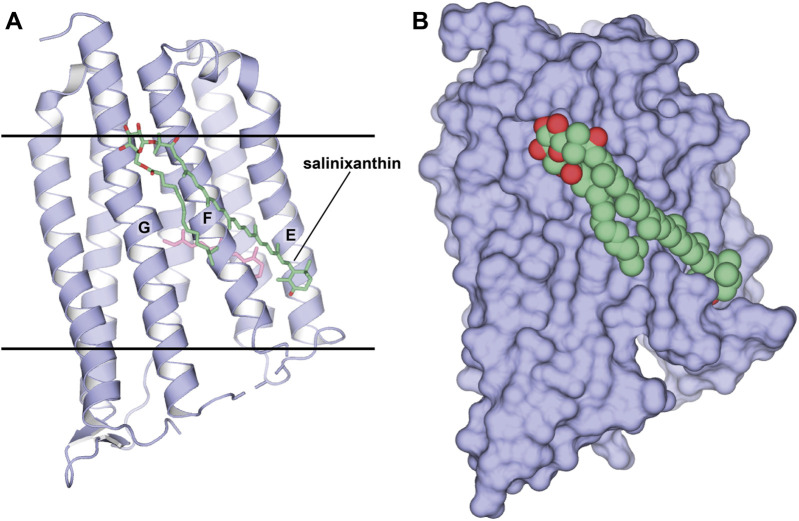
Structure of XR in a complex with the second chromophore. **(A, B)**, Structure, obtained from bicelles crystallization [PDB ID: 3DDL (Luecke et al., 2008)], is shown as cartoon and surface, respectively. The second chromophore, salinixanthin, is shown as sticks and spheres, correspondingly. **(A)**, Calculated hydrophobic-hydrophilic boundaries (Lomize et al., 2012) are shown with lines.

When the method was applied to Xanthorhodopsin (XR) (Luecke et al., 2008; Balashov et al., 2005), a bacterial-like light-driven proton pump obtained from the natural source, *S. ruber*, the second carotenoid chromophore named salinixanthin was preserved despite protein monomerization (1.9 Å-resolution, P1 space group, 2 molecules in ASU, PDB ID: 3DDL; Figure 5). It is likely that such a strong affinity is related to the functional importance of the second antenna for the protein. This is also evident by the fact that the antenna is located in the grooves on the protein surface. Besides salinixanthin, several alkyl fragments of two phospholipids are reported in the structure. While these are most likely dimyristoyl phosphatidyl-choline (DMPC) molecules from the bicelles, they occupy the grooves intended for structurally similar bacterial lipids.

### Membrane fusion crystallization

#### *Hs*BR lipids in the structures obtained with the membrane fusion method

**FIGURE 6:**
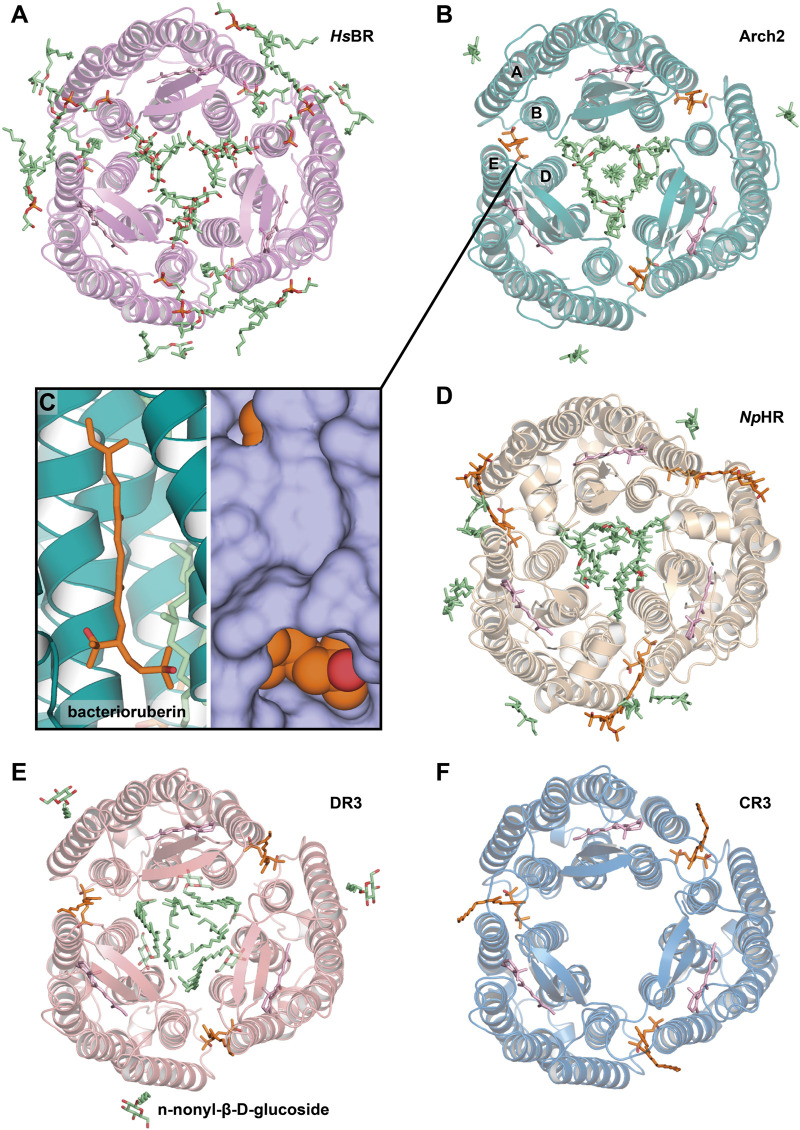
Lipids, resolved with membrane fusion method of crystallization. **(A)**, *Hs*BR [PDB ID: 1QM8 (Takeda et al., 1998)] **(B)**, Arch2 [PDB ID: 3WQJ (Kouyama et al., 2014)]. Bacterioruberin molecule is colored orange, and its binding site between helices A and B of one protomer and D and E of another is shown closer on the figure **(C)**. **(D)**, *Np*HR [PDB ID: 3A7K (Kouyama et al., 2010)] **(E)**, DR3 [PDB ID: 4FBZ (Zhang et al., 2013)] **(F)**, CR3 [PDB ID: 4L35 (Chan et al., 2014)]. All the structures are shown from the extracellular side.

*Hs*BR can be crystallized from the vesicles that are formed after incubation of the delipidizated PM with detergent at high temperatures [the Membrane fusion method (Takeda et al., 1998)]. The resultant vesicles are then used for standard vapor diffusion crystallization. The crystals of the P622 space group [Figure 6A, PDB ID: 1BM1 (Sato et al., 1999)] diffracted to 3.5 Å-resolution [which was then optimized to 2.5 Å, PDB ID: 1QM8 (Takeda et al., 1998), and, finally, to 2.3 Å, PDB ID: 1IW6 (Matsui et al., 2002)]. In each 2D membrane-like layer of the crystal, the *Hs*BR trimers are arranged in a honeycomb lattice. The *Hs*BR trimers in the crystal are glued by an unidentified archaeal lipid with a phytanyl chain between the helices A and B of one protomer and D and E helices of the adjacent protomer, as was observed for the benzamidine-crystallized *Hs*BR. The crystal packing analysis reveals that unlike for the benzamidine-crystallized *Hs*BR, the polar head of this lipid in the membrane fusion structure is stabilized by H-bonds with the mainchain of S35 and the sidechain of S35 and K40, all belonging to the AB-loop. At the same time, there are H-bonds with the sidechains of T107 (cytoplasmic part of helix D) and Y147 and K159 (cytoplasmic part of helix E) of the *Hs*BR from the other membrane layer. Such stabilization of the polar head explains why the authors saw a much more significant portion of the lipid compared to the benzamidine-crystallized protein. The authors also observed S-TGA-1 lipid in the inner compartment of the trimer from the extracellular side. Multiple other uncharacterized archaeal lipids were revealed on the surface of the trimer. The authors argue that the method reveals more lipids as it allows to avoid the solubilization of *Hs*BR but rather introduces only some detergent molecules to the native PM. Notably, the authors did not observe any lipids in the inner compartment of the trimers from the cytoplasmic site. According to them, this could be due to the replacement of the lipids by detergent.

#### Lipids of Archaerhodopsins

The membrane fusion method was then applied to study Archaerhodopsin-1 (Arch1) and Archaerhodopsin-2 (Arch2) ― close homologs of *Hs*BR from *Halorubrum* sp. *aus-1* and *-2*, respectively (Enami et al., 2006). Arch1 and Arch2 crystals (PDB ID: 1UAZ and 1VGO, respectively) were correspondingly in P4_3_2_1_2 and C222_1_ space groups, diffracted to 3.4 and 2.5 Å, and both contained two proteins in ASU with different conformations. Unlike *Hs*BR, both proteins lost their native trimeric organization in the crystals. This could be due to the utilization of high detergent concentration (the detergent:protein weight ratio was around 1:1 in both) that might have displaced the native lipids (which was not the case for *Hs*BR, obtained with a similar protocol). This is also evident by the presence of 12 n-nonyl-β-D-glucoside molecules in Arch2 (it should be noted that the limited resolution prohibits characterization of detergent molecules in the Arch1 structure). Thus, the harsh crystallization of Arch1 and Arch2 by detergent likely caused the dissociation of the oligomers.

In order to preserve the native structure, the protocol established for *Hs*BR was modified (Yoshimura and Kouyama, 2008). Instead of complete delipidization with high detergent concentrations, for new crystallization attempts the authors used native or partially delipidizated membrane. These crystallization attempts resulted in two structures of Arch2: in P321 (2.1 Å-resolution, 1 molecule in ASU, PDB ID: 2EI4) and P6_3_ space group (2.5 Å-resolution, 4 molecules in ASU, PDB ID: 2Z55). These structures (Figure 6B) both contained Arch2 in trimeric form, where native bacterioruberin molecules held together helices A and B from one protomer and D and E from another (Figure 6C). This bacterioruberin molecule likely stabilizes the oligomeric structure of Arch2. Notably, besides the bacterioruberin molecule, the authors resolved a lipid with a structure and location similar to S-TGA-1 lipid in *Hs*BR structures (in the inner compartment of the trimer, near helices B and C and BC-loop), which might also participate in the trimer stabilization.

More insights into Arch2-lipid interactions are provided by a 1.8 Å-resolution structure (Kouyama et al., 2014). The structure was interpreted in the H32 space group (1 protein molecule in ASU, PDB ID: 3WQJ). Besides the bacterioruberin molecule, it contained a hydrocarbon chain at the symmetrical center of the trimer that could be a squalene molecule, according to the authors. Important to note that the authors discussed the influence of the native lipid environment and oligomeric state on the protein’s function. Compared to the C222_1_ delipidizated monomeric structure of Arch2, in all the trimeric structures, a conserved R87 residue is facing towards RSB that is necessary for the correct formation of the proton transporting chain of H-bonds (Borshchevskiy et al., 2022). In contrast, in the monomer of Arch2, the R87 sidechain is directed towards the proton release group, which reminds the configuration observed for Arch3 (that was also crystallized in monomeric form) (Bada Juarez et al., 2021). The authors argue that the correct configuration is achieved when the extracellular end of helix C interacts with the diphytanyl group of the native lipid that fills the central opening of the Arch2 trimer.

#### Halorhodopsin from *Natronomonas pharaonis*

The light-driven chloride pump, halorhodopsin from *Natronomonas pharaonis* (*Np*HR), was expressed in the native expression system (Kouyama et al., 2010). A considerable fraction of bacterioruberin was removed by washing the claret vesicles with the 0.5% Tween-20 detergent, yet a considerable amount remained even after several washing cycles. The absorption spectrum of the claret vesicles had characteristic vibronic bands of bacterioruberin with peaks at 475, 504, and 540 nm and a broad band of retinal with a peak at 578 nm. Spectrum from the crystals confirms that approximately one bacterioruberin molecule is bound to one *Np*HR.

The crystals (PDB ID: 3A7K) showed diffraction to 2 Å, were in the C2 space group, and contained three protein molecules in ASU (Figure 6D). The protein was in the correct trimeric oligomeric state with one bacterioruberin bound in the usual location between the protomers. However, besides bacterioruberin, the structure possesses a large number of other lipids, participating in the stabilization of trimers and crystal packing. Interestingly, one of the unidentified lipids is located close to the bacterioruberin molecules, indicating that it might also participate in the stabilization of the latter in the structure. The central compartment of the trimer is filled with lipid molecules: the extracellular part (where S-TGA-1 lipid is bound in *Hs*BR) has three phospholipids, while the cytoplasmic part is filled with seven unidentified lipids. The latter, according to the authors, could be some specific lipids, like cardiolipins, which break the local 3-fold symmetry, similarly to what was observed for *Hs*BR and Arch2. Finally, the authors discuss the possibility that the halorhodopsin from *H. salinarum* (*Hs*HR; P6_3_22 space group, 1.8 Å-resolution, 1 molecule in ASU, PDB ID: 1E12), previously solved using LCP crystallization (Kolbe et al., 2000), has a different quaternary structure compared to *Np*HR because of the absence of the native lipids in the former. They argue that the protomers in *Hs*HR are rearranged to compensate for the absence of the native lipids that should have been accommodated in the inter-subunit crevices.

#### Deltarhodopsin-3 from *Haloterrigena thermotolerans*

Deltarhodopsin-3 from *Haloterrigena thermotolerans* (DR3) was expressed in *H. salinarum*, by introducing DR3-carrying plasmid to *Hs*BR-deficient strain MPK409 (Zhang et al., 2013). The claret membrane had characteristic peaks at 475, 505, and 541 nm, which is attributed to the presence of the second chromophore bacterioruberin. The protein was crystallized with the membrane fusion method, where the delipidization step of the membrane by the Tween-20 detergent was omitted, and relatively higher detergent concentrations were used. The crystallization at least partially preserved bacterioruberin, as indicated by the *in crystallo* absorption spectrum.

The crystals (PDB ID: 4FBZ) were in the R32 space group, diffracted to 2.7 Å and contained 1 molecule in ASU (Figure 6E). Between the protomers, besides expected bacterioruberin, the authors detected a squalene molecule sandwiched between helix C of one subunit and helix D of an adjacent subunit (located differently, compared to that one observed in *Hs*BR). The presence of an additional squalene molecule can explain why the protein has a lower affinity to bacterioruberin than Arch2, as the former might partially compensate for the trimer stabilization function.

The authors note that the resultant trimer of DR3 has larger than in *Hs*BR size of the cytoplasmic cross-section, meaning that lipids occupying the central opening of the trimeric structure of DR3 are different from those in the *Hs*BR trimer. They also point out that *H. thermotolerans* do not produce S-TGA-1 lipids, which is why they were not observed in the structure of DR3, even though the protein was expressed in *H. salinarum*. Finally, the authors hypothesize that the protein surface can partially adapt to the lipids of the host organism. From our side, we would add that the protein might also choose the most suitable lipids available.

#### Cruxrhodopsin-3 from *Haloarcula vallismortis*

Cruxrhodopsin-3 from *H. vallismortis* (CR3) was expressed in *H. salinarum* MPK409 similarly to the protocol described for DR3 and then crystallized with the membrane fusion method (Chan et al., 2014). As for DR3, the authors observed characteristic peaks for bacterioruberin in the spectra of both CR3-rich claret membrane and CR3 crystals. The crystals of CR3 trimers were grown in pH 4 and 5 (PDB ID: 4JR8 and 4L35, respectively), were both in the P321 space group, diffracted to 2.3 Å and 2.1 Å, respectively, and contained 1 molecule in ASU each. Each structure has a bacterioruberin molecule bound in the similar position described above (Figure 6F). The authors showed the importance of the bacterioruberin molecule for stabilizing the trimeric structure of CR3 in high detergent concentrations, which in turn protects the protein from photobleaching. As a result, CR3 was more stable than *Hs*BR, where no bacterioruberin molecule was observed.

### What happens with lipid-MP interaction when archaeal lipids are completely replaced with bacterial ones

In all cases described above, the MRs were expressed in native or close to native systems. One can still assume that native lipids were preserved for all methods, and the observed structural differences are method-specific artifacts. For example, some methods may yield crystals in which the position of certain lipids is fixed by crystal contacts. It would be interesting then to consider cases where archaeal MRs were heterologously expressed in bacteria, which completely excludes such a possibility.

#### Case of *E. coli* expression of *Hs*BR

Lipids in bacteria have alkyl sidechains instead of archaeal phytanyl sidechains, which could affect *Hs*BR trimeric assembly and influence protein function. To probe the influence of bacterial lipids on *Hs*BR structure, we heterologously expressed the protein in *E. coli* and crystallized it in LCP, using the same protocol as for *H. salinarum* protein (Borshchevskiy et al., 2022). The crystals [PDB ID: 4XXJ (Bratanov et al., 2015)] diffracted to 1.9 Å-resolution, were in the C2 space group and contained a trimer in ASU. Moreover, the trimers in the membrane layers were packed similarly to native PM and P6_3_ crystals grown *in meso* from PMs. No native *Hs*BR lipids were detected in the structure. However, it is remarkable that the resolved lipid chains (bacterial or MO from LCP) were located in the same clefts intended for the phytanyl sidechain, as they were trying to substitute and imitate the native lipids.

**FIGURE 7:**
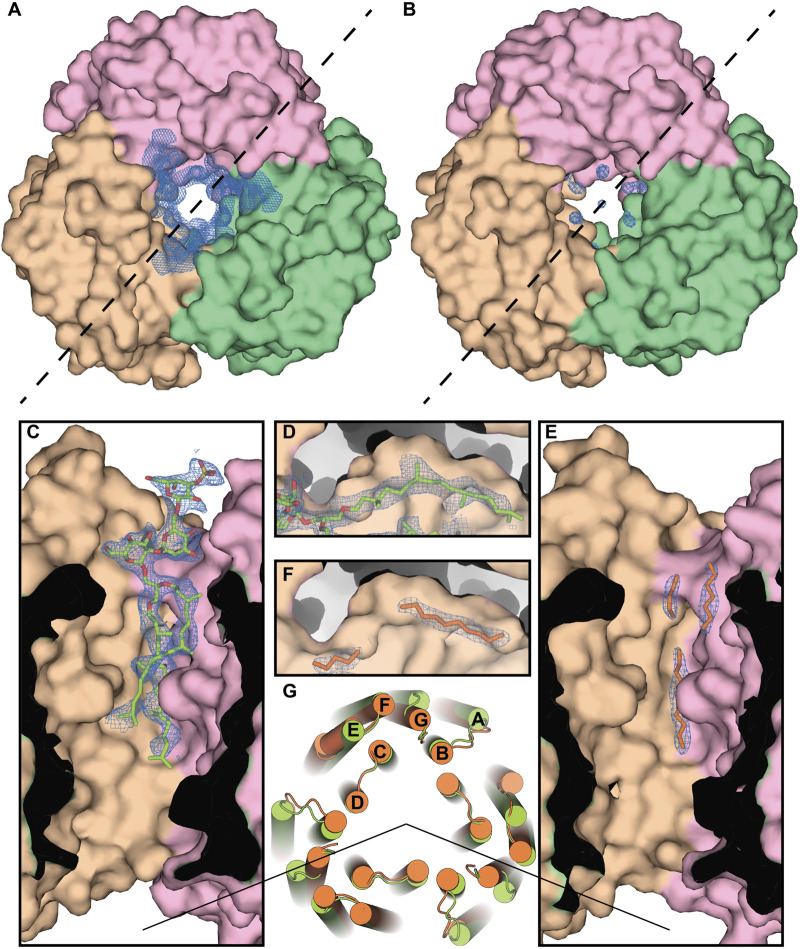
Specificity of the lipid-MP interactions in *Hs*BR. **(A)**, Crystal structure of the ground state of *Hs*BR expressed in native archaea [PDB ID: 1IW6 (Matsui et al., 2002)], a view from the extracellular side. In the panel, we show the 2*m*Fo-*D*Fc maps around the lipid in the central compartment taken from the structure of the M state of *Hs*BR [PDB ID: 2ZZL (Yamamoto et al., 2009)] due to the absence in the PDB of structural factors for the ground state structure. The trimer was generated by applying symmetry operators to the *Hs*BR molecule. **(B)**, Crystal structure of the ground state of *E. coli*-expressed *Hs*BR [PDB ID: 4XXJ (Bratanov et al., 2015)]. Chains A, B, and C of the protein are colored pink, brown, and green, respectively. **(C, E)**, Section for both structures by the dashed line, correspondingly. Electron density (contoured at 1 r.m.s. level) unambiguously shows the purple membrane S-TGA-1 lipid (colored light green) in the *H. salinarum*-expressed structure. In the case of the *E. coli*-expressed protein, linear carbohydrates of the anticipated bacterial lipids (colored orange) mimicked the glycolipid only partly, resulting in discontinuous electron densities (contoured at 1 r.m.s. level). **(D, F)**, The protein has the cavity, intended for the isoprenoid lipids, and the methyl-branched group of S-TGA-1 is facing towards it in the *H. salinarum*-expressed protein. The absence of methyl-branching in the bacterial lipids presumably leads to the local chain disorder in the *E. coli*-expressed protein. **(G)**, Differences in the positioning of the protein alpha helices in the cytoplasmic part (*H. salinarum*- and *E. coli*-expressed ground state structures are colored light green and orange, correspondingly), apparently due to the different lipid composition.

As an example, we show the central compartment of the periplasmic part of the protein (Figure 7). The protein has a cleft intended for the isoprenoid lipids, and the methyl-branched group of S-TGA-1 is facing toward it in the *H. salinarum* structure [PDB ID: 1IW6 (Matsui et al., 2002)]. However, in the case of *Hs*BR expressed in *E. coli*, linear carbohydrates of the putative bacterial lipids mimic the absent glycolipid only partially, resulting in discontinuous electron densities. The absence of methyl-branching in the bacterial lipids presumably leads to less tight contact with the *Hs*BR surface, resulting in the local chain disorder in the obtained structure. This is also reflected in a slightly larger average distance between the atoms of non-native lipids and the atoms of the hydrophobic surface of the protein. For the three alkyl chains that replace S-TGA-1 lipid in the *E. coli*-expressed *Hs*BR (LFA301 and LFA302 from chain A and LFA303 from chain C; 25 atoms in total), the average distance is 3.98 ± 0.35 Å, while for the atoms of the S-TGA-1 lipid taken at the same positions (O_2_, C_2_, C_3_, C_12_-C_21_, C_41_-C_45_, C_47_-C_60_ atoms of L2P270 from chain A; 32 atoms in total), it is 3.83 ± 0.40 Å. The difference is small, but this is not surprising ― the atoms of non-native lipids that are located far from the hydrophobic surface due to non-optimal geometry will be disordered. Although this turned out to be true for the particular example of the S-TGA-1 lipid, we believe that a more detailed study of this issue is necessary using other lipids and all the structures.

Important differences in the positioning of the protein alpha helices in the cytoplasmic part, apparently due to the different lipid composition, are clearly visible. Both structures can be superimposed with helical Cα r.m.s.d. (r.m.s.d., calculated over Cα atoms of the helices) of 1.7 Å, which can be accounted for the terminal region flexibility and difference in crystal packing. However, in chain B of the *E. coli* trimer, the C terminus of the E helix (T157-E161) is disordered. We anticipate that this might be connected with the lipid composition of the crystal. The disorder was previously observed for the solubilized *Hs*BR with no detectable S-TGA-1 densities in the central compartment [PDB ID: 3NS0 (Borshchevskiy et al., 2011) or 4MD2 (Borshchevskiy et al., 2014)], but not in the cases where the lipids were unambiguously observed and confirmed by MS data [PDB ID: 1BRR (Essen et al., 1998) or 1QHJ (Belrhali et al., 1999)].

#### *Haloarcula marismortui* bacteriorhodopsin I

Another important case is the crystal structure of *Haloarcula marismortui* bacteriorhodopsin I [*Hm*BR, PDB ID: 4PXK (Shevchenko et al., 2014)]. The crystals were diffracting to 2.5 Å-resolution, were in the P321 space group, and contained 1 molecule in ASU. As *Hs*BR, *Hm*BR was heterologously expressed in *E. coli*, where native archaeal lipids are absent. Nevertheless, the protein was successfully crystallized *in meso* in the preserved trimeric form. Moreover, we point out that the LCP host lipids fastened the trimeric form of *Hm*BR in a similar way that archaeal lipids wrap *Hs*BR homologs that were expressed in the corresponding native systems (Yoshimura and Kouyama, 2008; Takeda et al., 1998; Essen et al., 1998; Luecke et al., 1999; Belrhali et al., 1999; Zhang et al., 2013). Thus, again, one can see that non-native lipids are trying to compensate the native ones by binding to the same clefts.

#### *Haloquadratum walsbyi* bacteriorhodopsin

Finally, we should mention structural studies of *Haloquadratum walsbyi* bacteriorhodopsin (*Hw*BR). This acid-resistant homolog of *Hs*BR was also expressed in *E. coli* and crystallized using the *in meso* approach (Hsu et al., 2015). This resulted in two structures: a trimer (1.85 Å-resolution, C2 space group, PDB ID: 4QI1) and an anti-parallel dimer (2.57 Å-resolution, C2 space group, PDB ID: 4QID). Both structures contain unassigned lipids surrounding the protein. Interestingly, four lipids in total in the trimeric structure are located between the protomers, pointing out their role in its stabilization. The LCP structure of the trimeric protein obtained by the other group showed similarly located stabilization lipids [2.13 Å-resolution, C2 space group, PDB ID: 5KKH (Broecker et al., 2016)].

In the two previous cases (*Hs*BR and *Hm*BR), the structure was obtained only in the trimeric form, which may indicate the stability of trimers regardless of the lipid environment. However, in the case of *Hw*BR, we see that the trimeric structure can be disrupted, probably due to the loss of stabilizing lipids. A natural question arises: can any of the *E. coli* lipids perform a stabilizing function for the trimers? If so, then crystallization conditions can probably knock out these lipids, which leads to a dimeric structure. In this regard, the paper by Broecker et al. (2017) seems very informative. In the work, the styrene-maleic acid (SMA) copolymers were used to extract *Hw*BR with their lipids right from the *E. coli* membrane and then use the resultant nanodiscs for the LCP crystallization. Two structures were obtained: a trimer of the protein from detergent (2.18 Å-resolution, C2 space group, PDB ID: 5ITE) and a trimer from SMA nanodiscs (2 Å-resolution, C2 space group, PDB ID: 5ITC). Both structures possess unidentified stabilizing lipids between the protomers in similar locations. For this reason, the authors conclude that the bacterial phospholipids, due to their non-specificity to the archaeal protein, might have been displaced by MO from the LCP, and these MO stabilize trimers by filling empty spaces between protomers. While this can be true, still, two questions remain: (1) why, in certain conditions, does *Hw*BR form anti-parallel dimers if LCP lipids should have replaced bacterial ones in both cases (could it be due to the utilization of PEG 200 in crystallization conditions?) (2) why does tmBR [triple mutant of *Hs*BR, T17A/T24A/T47A, in which all mutations are located on the protein surface (Melnikov et al., 2022)] exclusively grow as anti-parallel dimers, while being oligomers in detergent? We believe that it would be useful to study in more detail which lipids predominate around heterologously expressed archaeal proteins: bacterial or LCP ones.

### Noble gases as a probe for protein-lipid interactions

Now, when we see how lipids can affect both the structure and function of MRs, there is a need to be able to probe these interactions. In the next part, we will consider noble gas derivatization as an instrument that can be used systematically to study lipid-MP interactions.

#### Noble gases can bind to the lipid binding sites

High-pressure derivatization was previously used to study *Hs*BR dynamics between the M and N states [PDB ID: 2ZFE (Hayakawa et al., 2008)]. It was shown that noble gases (xenon and krypton) could occupy hydrophobic cavities on the MP surface between helices C and D. It was concluded that this helps water molecules to enter the cytoplasmic part in the N state to reprotonate RSB from D96.

**FIGURE 8:**
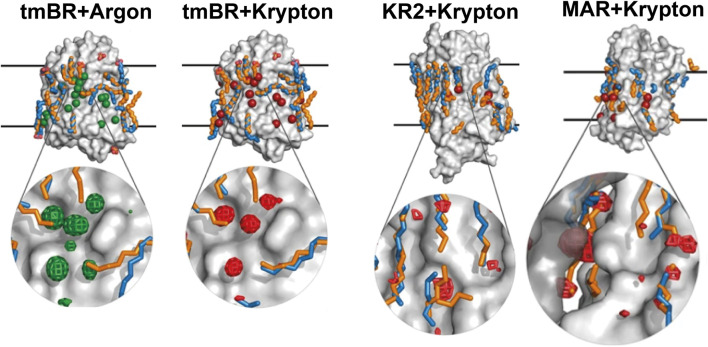
Noble gas positions on the surfaces of the derivatized structures of MRs. The data for tmBR + Argon, tmBR + Krypton, KR2+Krypton, and MAR + Krypton complexes is taken from the deposited PDB structures (Melnikov et al., 2022): 7Q38, 7Q35, 7Q36, and 7Q37, correspondingly. Calculated hydrophobic-hydrophilic boundaries (Lomize et al., 2012) are shown as black lines. Krypton and argon atoms are shown as red and green spheres, respectively. Lipid fragments present in the derivatized structures are shown in sky-blue; lipid fragments from native structures are shown in orange for comparison. Examples of noble gas binding sites are shown under magnification, where anomalous difference densities of krypton (red grid) and argon (green grid) are contoured at the 3.0 r.m.s. level. Figure adapted from Melnikov et al. (2022).

Our recent work (Melnikov et al., 2022) was conducted at much higher pressure [2,000 bar for argon, compared to 10 bar used in Hayakawa et al. (2008)] with argon and krypton demonstrating their ability to bind to the protein surface displacing lipids (Figure 8). Although noble gases are traditionally seen as chemically inert, the study demonstrates their ability to bind to the hydrophobic surface of MRs, displacing lipids, and possibly influencing protein dynamics. The study was framed within the context of anesthetics; however, an important question is posed (anesthesia being very much related to) about the role of lipids in the structural stability of MPs and, therefore, in its inherent function. The displacement of lipid molecules by noble gases, an unexpected but significant result, suggests that these gases may alter lipid-MP interactions. The results, particularly regarding lipid displacement and dynamics suppression, open avenues for further exploration into how lipids affect MP function via van der Waals interactions.

We speculate that noble gas atoms may imitate the native lipid environment because their symmetric shape allows them to bind all across the MP surface landscape as the native lipids are supposed to do. To be noted, though, that this effect might be exaggerated by noble gas atoms, which are more flexible, compared to lipids with their geometric constraints, in where to bind on the MP surface. Thus, the obvious difference might be that native lipids stabilize a MP just enough for proper dynamics in MP’s working range, while noble gas atoms, if at high concentration, might reduce the MP’s dynamics by collective van der Waals stabilization so that the MP is unlikely to adopt its full native conformational ensemble.

#### Molecular dynamics simulations in the studies of lipid-MP interactions

A major specificity of our previous work is its comprehensive use of crystallography and molecular dynamics (MD) simulations to map noble gas binding sites, with each technique having its own advantages and limitations. The crystallographic structures, although they are real experimental data, do not shed full light on the underlying objects because crystals are far from protein native conditions (i.e., a lot of noble gas atoms were found in between the crystal contacts of the neighboring molecules–places in which they might not bind when on a single molecule); moreover, crystallization lipids are primarily designed not to accommodate a particular MP in them, but to create a mesophase with a specific curvature. While the MD technique is completely an *in silico* method, it can give an insight into how noble gas atoms get arranged on the hydrophobic surface of a single MP molecule in a plain simulated lipid bilayer.

**FIGURE 9:**
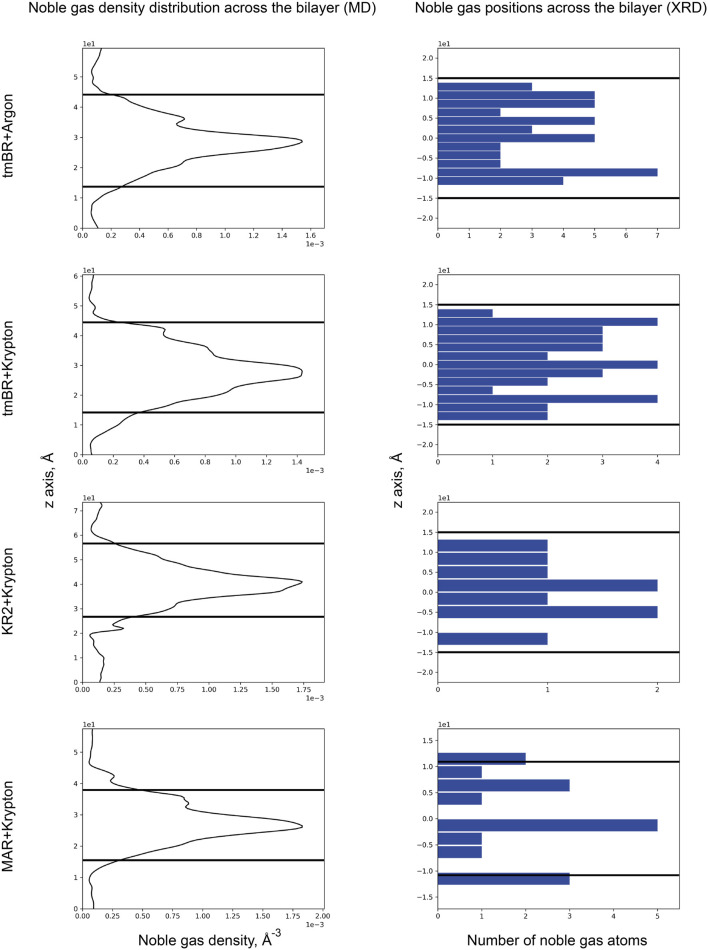
Noble gas distribution in four MR structures across the bilayer: (top to bottom) tmBR + Argon, tmBR + Krypton, KR2 + Krypton, and MAR + Krypton. Left part: Distribution of noble gas density in the direction perpendicular to the bilayer in the MD experiment. Data is taken from Melnikov et al. (2022). Right part: Distribution of noble gas positions in the refined crystallographic structures in the direction perpendicular to the bilayer. The data is taken from the deposited PDB structures (Melnikov et al., 2022): 7Q38, 7Q35, 7Q36, and 7Q37, correspondingly. In the MD experiment, the origin of a z-axis was at the calculation box’s lower boundary, while for the crystal structures, the origin of the z-axis was taken at the median of the bilayer. Thick black lines indicate the positions of the calculated hydrophobic-hydrophilic boundaries (Lomize et al., 2012).

In the current work, based on previous data, we have conducted an analysis of the positions of noble gas atoms relative to the bilayer depth. As can be seen from the MD simulation results for 3 MRs: tmBR, KR2, and MAR (Figure 9, left part), noble gas atoms tended to concentrate on the hydrophobic surface of proteins in the core of the bilayer. However, in the crystal structures, we do not see such a prevalence of noble gas in the hydrophobic core. According to the available data (which seems to be of poor statistical significance, at least for KR2 and MAR structures), noble gas atoms tend to occupy positions in the membrane halfway to the membrane interface from both sides of the hydrophobic core. It is demonstrated by the three peaks in the histograms of noble gas positions–one for the core and two–from both sides of the core (Figure 9, right part). This result might also be biased by the presence of crystal contacts between protein molecules in which noble gas atoms are easily accommodated. Such binding sites might not be relevant for the proteins in the native environment.

**FIGURE 10:**
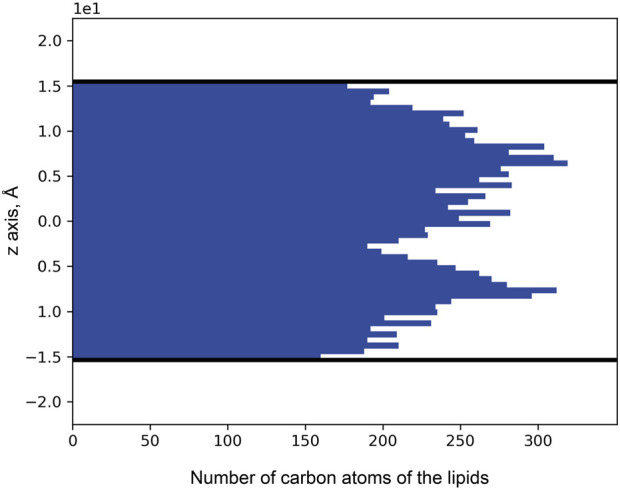
Distribution of lipid carbon atom positions across the bilayer depth (zero corresponds to the core of the bilayer). The positions were taken from the 71 unique MR structures that had been deposited in the PDB. Thick black lines indicate the positions of the calculated hydrophobic-hydrophilic boundaries (Lomize et al., 2012). Only atoms within the boundaries are taken into account.

Puzzled with the observed distribution of noble gas across the membrane, we decided to investigate the distribution of lipids. For that, we used 71 unique MR structures with a high-resolution limit above 2.0 Å available in the PDB. The MRs were aligned by the backbones and retinal molecules, after which the coordinates (in the normal to the membrane direction) of lipid atoms proximal to the main protein chain were extracted. As a result, we see two peaks in the histogram of binding position coordinates (Figure 10), which indicate that lipids tend to bind the protein surface tighter in the mid-region of the membrane, between the membrane interface and its core. The data indicates that most of the lipid fragments we are able to resolve and more specificity in lipid-MP interactions will be in these regions of MPs.

## Discussion

In the present work, we have analyzed a number of available crystal structures of MRs. The structures show that the lipids nearest to the MR surface strongly interact with them, supporting the idea of the existence and functional importance of annular lipids (Gómez-Fernández and Goñi, 2022). The hydrocarbon chains of such lipids are usually placed along the groves on the MR surface. This provides stronger binding of the lipids to MRs due to a larger area for the van der Waals interaction.

In most cases, the polar heads of these lipids are not visible due to the flexibility of the links between polar heads and hydrocarbon chains. Therefore, it is often a challenge to identify the type of lipids and to draw a conclusion on their importance for native MR assemblies. Nevertheless, in some crystallographic structures of MRs, several lipids were unambiguously identified. Often such lipids are placed between the protomers of the oligomers, playing a crucial role in their stabilization. This was demonstrated in the example of bacterioruberin molecules that, by interacting with helices from the adjacent protomers of archaeal MRs, provide native oligomerization and functional configuration of the active site.

Also, the presented structures suggest that in some cases MRs are evolutionary predisposed to bind specific lipids. As an example, squalene and S-TGA-1 are observed in the structures of *Hs*BR. They are located in specific crevices, geometrically dedicated for them. Another example is the salinixanthin molecule in the XR structure. Bacterial or synthetic lipids and detergent molecules from the crystallization matrix try to fill these empty cavities in the absence of these native lipids. This was clearly demonstrated in the example of *Hs*BR, heterologously expressed in *E. coli*. In this case, linear chains of bacterial or LCP lipids are trying to compensate for the lack of methyl branching, specific to archaeal lipids, in the inner compartment of the trimer.

Next, we analyzed the lipids in the structures of MRs, obtained by different crystallization approaches. We tried to understand which approach is better for studying native lipid-MPs complexes and how different approaches result in different compositions of MR hydrophobic surfaces. We conclude that crystallization from detergent micelles, LCP, and bicelles or using other artificial lipid-detergent systems (Faham and Bowie, 2002; Polovinkin et al., 2014; Borshchevskiy et al., 2010; Nikolaev et al., 2017) preserves only the most essential lipids with the highest affinity to MRs. At the same time, the membrane fusion method, in general, revealed much more native lipids. Unfortunately, the method requires high concentrations of MRs in the membranes and the softness of the latter (e.g., PM). This is a significant disadvantage of the approach compared to the universality of LCP crystallization. One of the possible solutions could be to add lipid extracts from the native organisms during the LCP crystallization [which, in small concentrations, would not disturb the phase (Cherezov et al., 2002)]. In the case of *Hs*BR, it was shown that LCP crystallization is possible without any solubilization at all, and the crystals thus obtained diffract at a sufficiently high level (3.5 Å-resolution) for studying native lipids (Nollert et al., 1999). Another idea that might work is the utilization of SMA polymers for the extraction of MRs with their native lipid environment right from the cellular membranes (Broecker et al., 2017). When protein production in the native organism is not available, the cell-free methods could work, where the expression system should be supplemented with natural lipids (Shimono et al., 2009; Furuse et al., 2015).

We discussed the crystallization approaches and suggested good practices that should be followed to get the best out of available. Yet, we would like to stress that despite very important information that one could extract from the structural data, this would not be sufficient to get a systematic picture of lipid-MP interactions. It is not a surprise. Indeed, nearly all the available structural studies did not aim to reveal the nature and the laws of lipid-MP interactions. In order to achieve this, the structural experiments should be planned from the beginning with the control of solubilization and crystallization conditions. The studies should be completed with functional (e.g., spectroscopic) studies, which are easy to do for MRs (Von Stetten et al., 2015; Engilberge et al., 2024; Efremov et al., 2006). Detergent concentrations must be controlled, with the methods like FTIR (DaCosta and Baenziger, 2003). Complementary studies with MS of native membranes, solubilized protein, and crystals are also necessary to reveal the nature of the lipids that are preserved at different stages of crystallization (Essen et al., 1998; Belrhali et al., 1999; Le and Loo, 2023). MD simulations are highly desirable, as they might help to avoid wrong conclusions arising, e.g., from the possible influence of crystal packing on the interactions (Inada et al., 2020; Chen et al., 2022; Enkavi et al., 2019; Muller et al., 2019). The MRs from different domains of life should be studied, and heterologous expression could be a very useful tool to understand patterns of lipid-MP interactions in different systems (Bratanov et al., 2015; Shevchenko et al., 2014).

Moving toward the analysis of such data, we should think about the standardized and correct modeling of lipids in the crystallographic structures. During our study, we found in the PDB at least 39 fragments (stored under 3 letter codes) that scientific groups use to model lipids in the crystal structures of MRs: 22B, 97N, ARC, C14, CPS, D10, D12, DAO, DD9, DPG, GLC, GOL, HEX, HP6, L2P, L3P, L4P, LFA, LI1, MAN, MPG, MYS, NAG, OCT, OLA, OLB, OLC, PCA, PCW, PH1, PLM, PX4, R16, SGA, SQL, SQU, SXN, TRD, UND. This makes the quantitative analysis of lipid-MP interactions difficult and incomplete, as there is no direct connection between the fragments and real lipids (at best, fragments can be combined into a lipid). The scientific community would profit if there was some kind of lipid database in structural studies, where all the related experimental data (like MS data) can be stored and connected to the PDB structures and the fragment codes. Also–more time should be devoted to correctly modeling the lipid electron densities, as there are currently many unmodelled density peaks or incorrectly modeled ones. We believe that by following these recommendations, progress in understanding lipid-MP interactions will not take long.

Although our work is exclusively devoted to the analysis of crystallographic structures, we should briefly mention cryo-EM structures, the number of which has increased significantly over the past years (Chazan et al., 2023; Podoliak et al., 2024; Shan et al., 2024; Hirschi et al., 2021; Hirschi et al., 2024; Rozenberg et al., 2022; Tucker et al., 2022; Kishi et al., 2022; Tajima et al., 2023; Morizumi et al., 2023; Tanaka et al., 2024). We see that despite the lower resolution of the structures, they often show high-quality densities from native lipids [see PDB ID: 6BAJ (Qiu et al., 2018) or 7N9Z (Li et al., 2021), and a detailed review by Valérie Biou (Biou, 2023)]. However, we believe that as more data accumulate, a similar study (on the effect of lipids on structure) should be performed with the cryo-EM structures. In particular, we are concerned that the nanodiscs into which proteins are often reconstituted for such studies may reduce the mobility of lipids at the MP surface. On one hand, this makes such lipids visible. On the other, this rigidity of lipids may not be native, affecting both the structure and function of the MPs.

**FIGURE 11:**
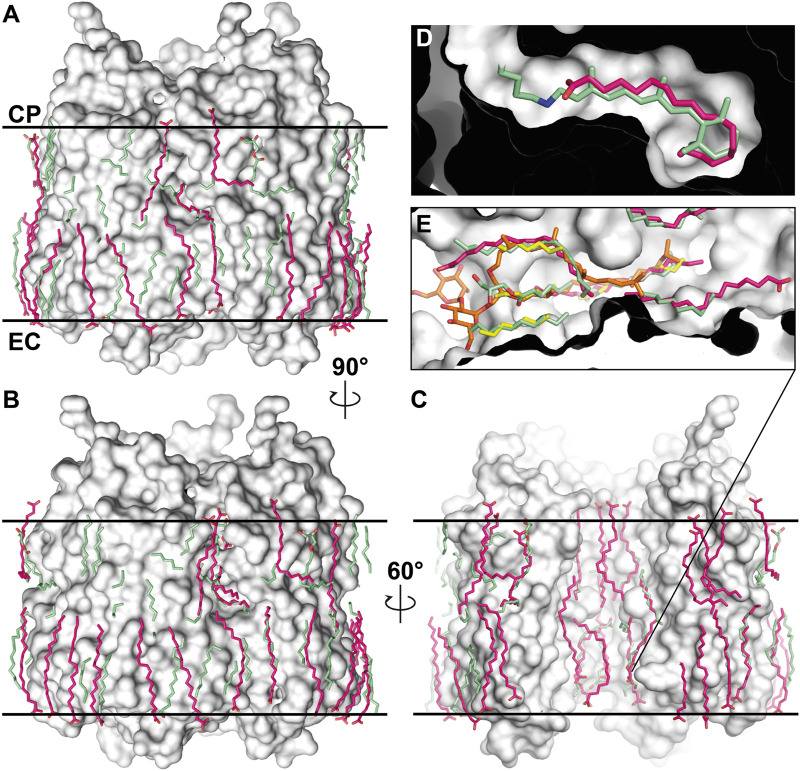
Distribution of lipids in the predicted structure of *Hs*BR. The prediction was conducted using the AlphaFold Server (https://alphafoldserver.com/) as described in the text. **(A–C)**, The predicted positions for 87 oleic acids (colored violet) are shown above the surface of the crystallographic *Hs*BR structure [PDB ID:7Z09 (Borshchevskiy et al., 2022), crystallographic lipids are colored green]. The structure is shown from three different angles. In figure **(C)**, one of the protomers was removed to reveal the inner compartment of the trimer. Thick black lines indicate the positions of the calculated hydrophobic-hydrophilic boundaries (Lomize et al., 2012). **(D)**, a single oleic acid molecule, consistently occupied the retinal binding site in each protomer of the predicted structures of *Hs*BR. **(E)**, predicted oleic acid molecules in the extracellular side of the inner compartment of the trimer are compensating for the absence of the native S-TGA-1 lipid [colored orange; coordinates for which were taken from S-TGA-1-bound structure of *Hs*BR, PDB ID: 1IW6 (Matsui et al., 2002)]. The same was observed in the crystal structure of *E. coli*-expressed *Hs*BR [colored yellow; PDB ID:4XXJ (Bratanov et al., 2015)].

With the rapid development of generative methods for structure determination, we certainly could not ignore AlphaFold 3 in our work (Abramson et al., 2024). The authors point out significant progress in predicting protein-ligand interactions. Although this is beyond the scope of the main work devoted to crystallographic data analysis, it was interesting to see how well the lipid positions are predicted for the *Hs*BR structure. For this, we used AlphaFold Server (https://alphafoldserver.com/) to generate the *Hs*BR trimers with 87 oleic acid molecules (as the closest analog of MO). The results are presented in Figures 11A–C. It is worth noting that at the time of preparing the manuscript, the server cannot generate the positions of archaeal lipids. The number 87 is since in the highest resolution structure of *Hs*BR available at the moment [PDB ID:7Z09 (Borshchevskiy et al., 2022)], 28 linear lipid densities per protomer were found, and three additional lipids required as they consistently occupy the retinal binding site in the generated structures (Figure 11D), which is especially interesting in connection with the recent work on proteorhodopsin biogenesis (Hirschi et al., 2024). As a result, we see that the lipid positions on the trimer surface are predicted poorly (also confirmed by the low plDDT score, which is lower than 50). However, interestingly, the lipids in the inner compartment of the trimer are predicted much better (50 < plDDT <70), and in general, as in the *E. coli*-expressed *Hs*BR structure [PDB ID: 4XXJ (Bratanov et al., 2015)], they try to compensate for the absence of the native S-TGA-1 lipid [PDB ID: 1IW6 (Matsui et al., 2002; Figure 11E)]. We see a great future for generative methods for studying lipid-MP interactions. For example, it will be possible to search for specific lipids for all MPs and understand how the absence of these lipids could affect the degradation of the native oligomers. However, to improve the performance of generative methods in predicting lipid positions, the model must be trained on structures with additional data on their lipid composition. Also, creating a benchmark to direct machine learning research efforts toward solving the problem at hand would be necessary. The distance to the hydrophobic surface (as we have calculated for the S-TGA-1 lipid) could be one of the metrics. Progress will be possible if we adhere to the recommendations for the experimental, processing, and annotation parts given above in the text.

Finally, we showed that the displacement of lipids by noble gases under high pressure could be used to probe such lipid-MP interactions and monitor the functional consequences of such changes (Melnikov et al., 2022). The structural data, accompanied by flash-photolysis data under pressure (Hayakawa et al., 2008), would reveal more information on the anesthetic effects of lipids and can become a part of a pipeline, probing lipid-MP interactions. However, before this happens, it will be necessary to learn to distinguish the effects associated with the displacement of lipids from other effects, such as the blocking of important sites by noble gases (Sauguet et al., 2016) or the deformation of MPs under pressure (Abiko et al., 2019; Abiko et al., 2022).

We want to summarize all the above-mentioned suggestions and speculations briefly. First, we hypothesize that to understand the molecular mechanisms of MPs, it is not sufficient to consider MPs themselves. The first layer of the lipidic belt, annular lipids, can also be essential for MP structure and function. Second, the MP surface and certain native lipids are evolutionarily selected to fit each other. Such lipids strongly bind to the crevices on the surface of MP, dedicated for them to provide strong van der Waals interaction. But these specific lipids should not make the MPs too rigid as this would suppress the dynamics of the latter, necessary for the correct functionality. Third, the function of MPs (besides dynamics) can be susceptible to the lipids at the MP surface, especially to the specific ones.

Reflecting on the results of this analysis, we want to speculate that lipid-MP “atomic mismatch” (due to mutations on the MP surface or errors during lipid biosynthesis) might cause some age-related diseases [e.g., Niemann–Pick Type C Disease (Wheeler et al., 2019); the various effects of lipids on MPs are well described in another review for further reading (Marsh, 2008)]. Certainly, systematic studies, partially discussed in our work, are necessary to verify these hypotheses and speculations.

## Data Availability

Publicly available datasets were analyzed in this study. This data can be found here: Protein Data Bank. Molecular dynamics simulations data and AlphaFold 3 predictions are available upon request.
